# Reducing Auditory Hypersensitivities in Autistic Spectrum Disorder: Preliminary Findings Evaluating the Listening Project Protocol

**DOI:** 10.3389/fped.2014.00080

**Published:** 2014-08-01

**Authors:** Stephen W. Porges, Olga V. Bazhenova, Elgiz Bal, Nancy Carlson, Yevgeniya Sorokin, Keri J. Heilman, Edwin H. Cook, Gregory F. Lewis

**Affiliations:** ^1^Department of Psychiatry, University of North Carolina at Chapel Hill, Chapel Hill, NC, USA; ^2^Department of Psychiatry, Institute for Juvenile Research, University of Illinois at Chicago, Chicago, IL, USA; ^3^Department of Occupational Therapy, Elizabethtown College, Elizabethtown, PA, USA

**Keywords:** autism, auditory hypersensitivities, social engagement behaviors, listening, polyvagal theory

## Abstract

Auditory hypersensitivities are a common feature of autism spectrum disorder (ASD). In the present study, the effectiveness of a novel intervention, the listening project protocol (LPP), was evaluated in two trials conducted with children diagnosed with ASD. LPP was developed to reduce auditory hypersensitivities. LPP is based on a theoretical “neural exercise” model that uses computer altered acoustic stimulation to recruit the neural regulation of middle ear muscles. Features of the intervention stimuli were informed by basic research in speech and hearing sciences that has identified the specific acoustic frequencies necessary to understand speech, which must pass through middle ear structures before being processed by other components of the auditory system. LPP was hypothesized to reduce auditory hypersensitivities by increasing the neural tone to the middle ear muscles to functionally dampen competing sounds in frequencies lower than human speech. The trials demonstrated that LPP, when contrasted to control conditions, selectively reduced auditory hypersensitivities. These findings are consistent with the polyvagal theory, which emphasizes the role of the middle ear muscles in social communication.

## Introduction

Frequently accompanying a diagnosis of autism spectrum disorder (ASD) are speech and language delays, difficulties in extracting human voice from background sounds, auditory hypersensitivities, and a general compromise in social communication skills (1–8). In contrast to the prevalent reports of auditory processing deficits, most individuals with ASD, even those with noticeable auditory perceptual disorders, have normal hearing when tested on a standard audiogram (9).

Several mechanisms have been proposed as contributing to frequently reported deficits in auditory processing including damage or dysfunction to peripheral structures (i.e., middle ear and inner ear), neural pathways (e.g., auditory nerve), and central structures (e.g., brainstem nuclei and cortical areas) (e.g., Ref. (10–17)). A review (18) suggests that although atypical auditory processing and both hypo- and hyper-reactivity to auditory signals are frequently observed in autism, these atypical reactions cannot reliably be attributed to specific neural pathways. Thus, subjective methods remain the sole indicators of auditory hypersensitivities (19).

### Physiology of the middle ear

Borg and Counter (20) described a role of middle ear muscles in facilitating the extraction of human speech by dampening the transmission of low frequency noise from the external environment to the inner ear. The Borg and Counter model suggests that atypical neural regulation of middle ear muscles may contribute to the frequently observed auditory hypersensitivities and auditory processing deficits in ASD. Deconstructing the path through which sound is processed illustrates the role middle ear structures have in auditory processing and how atypical neural regulation of the middle ear muscles may contribute to auditory hypersensitivities and atypical auditory processing.

Sound enters the outer ear and travels through the external auditory canal to the eardrum where it is transduced by the structures of the middle ear (i.e., small bones comprising the ossicular chain), which connects the eardrum with the cochlea. The rigidity of the ossicular chain determines the stiffness of the eardrum. The middle ear muscles, via cranial nerves, regulate the position of the ossicles and stiffen or loosen the eardrum. When the eardrum is “tightened” higher frequencies are absorbed and transmitted to the inner ear and the energy of lower frequencies is attenuated (i.e., reflected) before being encoded by the inner ear (cochlea) and transmitted via the auditory nerve (cranial nerve VIII) to the cortex. Complementing the ascending pathways are descending pathways that regulate the middle ear muscles, which functionally determine the energy (i.e., attenuate, pass, or amplify) of specific frequencies that reach the inner ear. The features describing the transformation of sound intensity from outer to inner ear defines the middle ear transfer function. If the acoustic information in the frequency band associated with speech is distorted by an atypical middle ear transfer function, the information being coded by the inner ear and subsequently being transmitted to the cortex will not contain sufficient information to enable accurate detection of speech sounds. In addition, there are descending pathways that regulate the hair cells in the cochlea to “fine tune” auditory perception, which is especially important in the development of language skills. If the acoustic information related to human speech that reaches the cortex via ascending pathways is distorted, then the descending pathways to the cochlea may also be atypical and will further distort the individual’s ability to process speech and to produce language.

As proposed by Borg and Counter (20), atypical central regulation of peripheral middle ear structures may pass low frequency sounds that dominate the acoustic spectrum in our mechanized society (e.g., ventilation systems, traffic, airplanes, vacuum cleaners, and other appliances) resulting in both a hypersensitivity to sounds and distorting or “masking” the frequency components associated with human speech reaching the brain. This emphasis on the functional role of the middle ear muscles in the dampening of background noise and the extraction of voice is based on a literature documenting two points: (1) the neural regulation of the middle ear muscles modulates the transfer function of the middle ear (21, 22) and (2) the transfer function of the middle ear determines the acoustic energy from low frequencies that reach the inner ear (23). Thus, an atypical middle ear transfer function would be a potentially parsimonious explanation of both the auditory hypersensitivities and the difficulties in auditory processing frequently associated with autism.

### Designing the listening project protocol

The listening project protocol (LPP) is a theoretical departure from the disciplines frequently involved in the treatment of auditory processing disorders, which emphasize the role of central structures in the processing of speech (see Ref. (18) for a review). LPP was theoretically designed to reduce auditory hypersensitivities by recruiting the anti-masking functions of the middle ear muscles to optimize the transfer function of the middle ear for the processing of human speech. LPP is based on an “exercise” model that uses computer altered acoustic stimulation to modulate the frequency band passed to the participant. The frequency characteristics of the acoustic stimulation were theoretically selected based on the documented frequency band and weights associated with the index of articulation (24) and speech intelligibility index (25). These indices emphasize the relative importance of specific frequencies in conveying the information embedded in human speech. During normal listening to human speech, via descending central mechanisms, the middle ear muscles contract and stiffen the ossicular chain. This process functionally removes most of the “masking” low frequency background sounds from the acoustic environment and allows human voice to be more effectively processed by higher brain structures. Modulation of the acoustic energy within the frequencies of human voice, similar to exaggerated vocal prosody, is hypothesized to recruit and modulate the neural regulation of the middle ear muscles and to functionally reduce auditory hypersensitivities (see Ref. (23)).

The features of the intervention including the context, the duration of stimulation, and frequency band selected were theoretically determined and based on the following neurophysiological principles: (a) the transfer function of the middle ear serves as an anti-masking mechanism to dampen low frequency sounds and to facilitate extraction of human voice from background sounds (20), (b) acoustic energy is readily transmitted across middle ear structures, regardless of the neural tone to the middle ear muscles, at a resonance frequency in children between 800 and 1200 Hz (26), (c) middle ear muscles are primarily composed of fast-twitch muscles and are vulnerable to rapid fatigue (27), and (d) the phylogenetic convergence in mammals of a brainstem area involved in the neural regulation of striated muscles of the face and head including the middle ear muscles (see (23, 28, 29)). Principles (a) and (b) were used to design the acoustic stimuli, principle (c) informed decisions related to the duration of each session, and principle (d) provided the basis for the social support provided during the intervention (i.e., the neural regulation of the middle ear muscles is optimized in a “safe” context).

LPP applies computer altered vocal music (i.e., filtered music) designed to exaggerate the features of human prosody and hypothetically to exercise the neural regulation of the middle ear muscles. By modulating the frequency band associated with human vocalizations, it was hypothesized that the ascending pathways would be providing dynamically changing information that would feedback on the descending pathways regulating the middle ear muscles. Metaphorically, the procedure could be conceptualized as a “treadmill” exercise for the middle ear muscles during which the demands to “listen” and process the acoustic features of the intervention stimuli were dynamically changing. To test the primary hypothesis that the filtered music condition would reduce hearing sensitivities in children with ASD, two trials were conducted. Trial I contrasted a filtered music group to a headphones only group and Trial II contrasted a filtered music group to an unfiltered music group.

The intervention consisted of five daily sessions of approximately 45 min during which the participant passively listened to the acoustic stimulation through headphones in a quiet room, while researchers provided social support to insure that the participants remained calm. The frequency bands were temporally modulated within each session and, independent of amplitude, the band of frequencies that were modulated progressively increased across the five sessions. Theoretically, the changing frequency bands were presented to increase the neural regulation of middle ear structures to dampen the perception of background low frequency sounds and to potentiate the extraction of human voice. Although middle ear muscle regulation could not be assessed, the Borg and Counter (20) model provided the scientific basis to hypothesize that the exercises embedded in LPP would reduce auditory hypersensitivities.

## Methods: Trial I and Trial II

### Participants

Potential participants contacted the laboratory for initial inclusion screening. Participants were informed about the research project by clinicians, parents who previously participated in our research program, and via professional presentations and/or newsletters. Individuals with a suspected diagnosis of ASD, who did not have a history of seizures, were scheduled for a diagnostic assessment that consisted of the autism diagnostic interview-revised (ADI-R) (30). The ADI-R provides a diagnostic algorithm consistent with the Diagnostic and Statistical Manual of Mental Disorders, fourth edition (DSM-IV) (31) and International Classification of Diseases, tenth edition (ICD-10) (32). Informed consent was obtained from parents. The Institutional Review Boards at the University of Maryland, the University of Illinois at Chicago, and the University of North Carolina approved the project. The protocols are excluded from the requirement to be registered (e.g., ClinicalTrials.gov), since enrollment was initiated before January 1, 2001 and data collection was completed before December 26, 2007.

Parents of 178 children contacted the laboratory to participate in the research. Based on the ADI-R criteria, 146 children met the full criteria of autism. Of the children, who did not meet full criteria, 29 exceeded the ADI-R cut off on at least the qualitative impairments in reciprocal social interaction and/or communication scales. Three children, who did not meet the cut off on either the qualitative impairments in reciprocal social interaction and/or communication scales, were excluded from participating in the research.

Based on presentation at the laboratory the first 73 children were assigned to Trial I. In Trial I, data from nine children (two in the filtered music and seven in the headphone only groups) were lost due to technical problems. In Trial I, questionnaire data were scored for 36 children in the filtered music group and 28 children in the headphones only group. Following the completion of Trial I, 102 children, who had not participated in Trial I, were enrolled in Trial II. In Trial II, due to scheduling difficulties, families of six children withdrew before participating in the trial and one family withdrew after the second day of the intervention. In Trial II, data from one child who was diagnosed with Fragile X were excluded from the data analyses. In addition, data from 12 children in the filtered music group were lost due to parents not returning the questionnaires, or returning the questionnaires late, or health issues. Data are not available for documenting the specific causes for lack of compliance. Questionnaire data in Trial II were available from 50 participants in the filtered music group and 32 participants in the unfiltered music group. Descriptive statistics of demographic features of the subjects from Trial I and Trial II with questionnaire data are reported in Table 1.

**Table 1:** **Demographic information for subjects with complete data by group assignment and sex**.

	Trial I		Trial II	
	Filtered music	Headphones only condition	Filtered music	Unfiltered music
	Mean age (SD)^b^	Mean age (SD)^b^	Mean age (SD)^b^	Mean age (SD)^b^
**Met at least partial criteria on ADI-R^a^**				
Male	58.24 (10.14), *n* = 25	49.46 (10.96), *n* = 23	54.89 (14.83), *n* = 44	56.20 (9.36), *n* = 27
Female^c^	48.67 (11.99), *n* = 11	61.00 (7.91), *n* = 5	44 (20.66), *n* = 6	60.33 (9.29), *n* = 5
**Total**	55.37 (11.42), *n* = 36	52.67 (11.30), *n* = 28	53.33 (15.95), *n* = 50	56.74 (9.25), *n* = 32

*^a^Exceeded the ADI-R cut off on at least the qualitative impairments in reciprocal social interaction and/or communication scales*.

*^b^Mean age and standard deviation in months*.

*^c^Females in Trial I were significantly older in the headphone only group*.

Trial I and Trial II included 86 participants in the filtered music condition, 32 participants in the unfiltered music condition, and 28 participants in the headphones only condition (see Table 1). Although mental age of the participants was not formally assessed, all participants had either speech (at least five words apart from “mama” and “dada,” used spontaneously and meaningfully) or followed verbal instructions. Approximately 80% of the participants were Caucasian and the remaining 20% included children from African–American, Latino, and Asian parents.

### Experimental design

The intervention research was conducted as two sequential randomized controlled trials with parallel control groups. All participants were randomly assigned sequentially by presentation at the laboratory to either the filtered music group or a control condition group. No clinical or behavioral feature was used to determine group assignment. Trial I participants were randomly assigned to either a filtered music or a headphones only group, which consisted of children wearing headphones without music.

Trial I was initiated to evaluate whether the intervention had an effect beyond the contextual variables of supportive play and low intensity social interactions that characterized the experimental environment for both groups. Since data analyses of parent questionnaires indicated a treatment effect on auditory hypersensitivities, Trial II was conducted to evaluate whether the filtering of the music uniquely determined intervention effects. Trial II participants were randomly assigned to either a filtered music group or an unfiltered music group. To insure a sample size sufficient to test hypotheses related to auditory hypersensitivities, twice as many participants were assigned to the filtered music group.

Parents were not informed about their child’s group assignment until the follow-up sessions were completed. Nor were parents informed about the features of the intervention (i.e., filtered music) or the control condition within each trial (i.e., headphones only in Trial I and unfiltered music in Trial II). Circumaural headphones were used, since they provide excellent sound quality, are comfortable to wear, and have excellent external noise rejection. The features of the headphone in combination with low intensity auditory stimuli precluded the parents from detecting whether their child was receiving the filtered music condition or a control condition. Based on our interactions with parents, it appeared that parents were not informed about the group assignment of their children. After the completion of the follow-up assessment sessions, the children in the unfiltered and the headphones only conditions were given the opportunity to receive the filtered music. Since knowing group assignment might bias parental perceptions of the child’s behavior, data from the children, who received the filtered music after participating in either the headphones only or unfiltered music conditions, were not included in the data analyses.

One week following the intervention, parent reports were obtained for all participants in both trials. None of the children who participated in Trial I participated in Trial II. In addition to the parent questionnaire, semi-structured play-based behavioral assessment sessions were conducted with the children and videotaped before and after the intervention.

### Conditions and procedure

Each condition (i.e., the filtered music, unfiltered music, and headphones only conditions) consisted of approximately 45 min sessions conducted during five-consecutive days. During the intervention, regardless of group assignment, each child wore headphones in the same laboratory environment. The same vocal music selections were used for both the filtered music and the unfiltered music conditions. In the filtered music condition, the vocal music was computer processed based on a proprietary algorithm developed to remove low and high frequencies and to modulate the width of the frequency band associated with human voice. The intervention stimuli were stored on compact discs and played via high quality compact disc player (Marantz CC-4000) to high quality over the ear headphones (Beyerdynamic DT831). Maximum loudness was calibrated at a peak of 75 dBC before the intervention started. During the headphones only condition, no auditory stimulation was provided through the headphones, although the context was identical to the filtered music and unfiltered music conditions. The low volume of the intervention stimuli and the use of over the ear headphones insured that the intervention stimuli could not be distinguished from the ambient background sounds in the test room by the parents.

The sessions were conducted in a research room with toys (e.g., books, doll house and accessories, parking garage and cars, pretend kitchen and accessories, stuffed animals, coloring books, and crayons). During the intervention, the children were able to freely play with the toys. One experimenter stayed in the room during the intervention to assist the child with the headphones when needed. Parents were also allowed to be in the room with their child. The experimenter and the parents were instructed to be quiet and to interact with the child only to maintain and to support a calm behavioral state. Due to the nature of the study (e.g., checking the integrity of the headphones), the experimenter who conducted the intervention session was not always blind to the child’s group assignment. In Trial I, since the headphones only group received headphones without sound, the experimenter was frequently aware of the child’s group assignment. However, since only the experimenter adjusted the headphones, the parents remained blind. In Trial II, since acoustic stimulation was being presented to both groups, the experimenter and the parent were unaware of the child’s group assignment. Accordingly, to avoid the possibility of rating bias, the experimenter who conducted the intervention sessions did not participate in the play-based assessments during which sharing behaviors were coded.

### Behavioral assessment

#### Parent questionnaire

Following the intervention and the play-based assessments, parents were given a structured questionnaire developed in our laboratory, targeting specific categories of their child’s developmental and behavioral problems including auditory hypersensitivities. The parents of children in all groups were instructed to complete and to return the questionnaire to the laboratory in a week. The questionnaire focused on whether the child had difficulties in a specific behavioral area and whether there were any changes in this area following participation in the research. For each behavioral category, parents were required to document changes, if any, following the intervention by providing specific examples of observed new behaviors. The structured questionnaire focused on the behavioral domains listed in Table 2.

**Table 2:** **Behavioral domains and explanations for the structured parent questionnaire**.

	**Definitions**
Hearing sensitivity	Exaggerated negative responses (e.g., crying or placing hands over the ears) to common noises (e.g., vacuum cleaner, garbage disposal, baby crying, and air conditioning)
Spontaneous speech	Non-prompted use of words and sentences to communicate thoughts and ideas
Receptive speech	Ability to understand instructions and phrases
Spontaneity	Non-prompted behaviors initiated by the child
Behavioral organization	Ability to occupy oneself (when left alone) in a productive and non-stereotypical way
Emotional control	Ability to calm quickly when upset, to respond to unexpected changes without getting upset, and to tolerate objections and contradictions of other people
Affection	Behaviors reflective of warm emotional state expressed by the child toward familiar people (e.g., hugging, kissing, and saying “I love you” to the parent)
Listening	Ability to focus on human speech without visual or contextual cues, to understand spoken words, and to follow verbal requests
Eye contact	Making and maintaining eye contact during social interactions
Relatedness	Non-prompted social behaviors that reflect understanding of a joint partnership in interactions and sharing the same goals during social interactions (e.g., looking at a partner, showing toys, sharing an idea or a thought, and directing emotions to the partner)

#### Questionnaire scoring

Each of the 10 items representing the behavioral domains described in Table 2 was scored as a 1, 0, or -1. A score of 1 was assigned if the parents indicated that their child had a problem in the area of interest before the participation in the project and provided an example of a new behavior that could be considered as an improvement in this area. An item received a score of 0 if the parents indicated that their child had a problem in the area of interest, but provided no example of a change. Non-specific parental responses (e.g., “somewhat better” and “a lot better”) that were not supported by concrete examples of the new behaviors also were conservatively scored as 0. An item received a score of -1 if the parent indicated that the behavior became worse after participating in the research and provided an example of the new worsened behavior. If the parent did not indicate a problem in the area of interest, the item did not receive a score. Each questionnaire was scored by two researchers, at least one of whom was blind to the child’s group assignment. Only when both scorers agreed that the example provided by the parent constituted a new and relevant behavior, a score of 1 was given. Scores of -1 were rare and did not occur on any of the behaviors coded in Trial I and only three times in Trial II. Thus, separate analyses for scores of -1s were not conducted.

#### Social interaction coding scale

Prior to and following their participation in the intervention project, all children participated in a 10-min semi-structured play-based observational assessment of social engagement skills with the social interaction coding scale (SICS) (33). The SICS provides information regarding the child’s social engagement activity. Similar to the autism diagnostic observational scale (ADOS) (34) and early social communication coding scales (ESCS) (35), the SICS requires a semi-structured presentation of standard tasks. Each task provides an opportunity for social engagement by requiring the child to engage in a joint activity. In the current study, the number of spontaneous sharing behaviors was quantified.

#### Coding social interaction coding scale

The frequency of sharing behaviors was coded from videotapes by trained coders. Coders obtained reliability with each other on training tapes before using the scale for research (i.e., 80% agreement on individual items, mean kappa > 0.60 for three consecutive joint scoring). Each tape was coded by two trained coders independently and compared for agreement. At least one of the coders was not aware of the participant’s group assignment when coding. Consensus was used to establish the final code. If raters disagreed on the same item, the code of the unbiased coder was recorded. If coders were uncertain about the final code, the opinion of the third trained coder was requested and the code that received the consensus of at least two coders was recorded. If all three coders disagreed on the final code, the behavior was not coded.

### Data analyses

Analyses of variance and non-parametric χ^2^ analyses were used to evaluate group differences within each trial on each of the behavioral domains. Since both analysis strategies identified the same group differences within each trial, only the analyses of variance are presented. A Bonferroni correction adjusted significance levels for multiple comparisons.

## Results

### Questionnaire data

#### Global evaluation of problems

Confirming the effectiveness of the randomization procedures, there were no group differences in the representation of the behavioral problems reported via the parental questionnaire within each trial or across trials (see Table 3). For example, the representation of hearing hypersensitivities across the four groups across both trials ranged from 43 to 50%. When the number of problem dimensions was summed for each participant, more than 95% of the parents reported that their child had at least one behavioral problem. The percentage of parents reporting multiple behavioral problems decreased as the number of domains increased, with approximately 80% of the parents reporting problems in at least five behavioral domains.

**Table 3:** **Distribution of initial behavioral problems (%) within each trial^a^**.

	Trial I		Trial II	
	Filtered music (%)	Headphones only group (%)	Filtered music (%)	Unfiltered music (%)
Hearing sensitivity	50	43	46	50
Affect	44	61	64	59
Eye contact	75	61	60	63
Behavioral organization	53	57	56	53
Emotional control	50	43	66	59
Spontaneous speech	75	82	82	78
Receptive speech	72	82	90	81
Listening	81	86	74	66
Spontaneity	69	71	44	44
Relatedness	83	82	64	66
At least 1 problem	92	96	98	97
At least 2 problems	92	93	98	94
At least 3 problems	89	89	96	91
At least 4 problems	83	79	94	88
At least 5 problems	81	75	92	78

*^a^No significant differences were found among the groups on any behavioral dimension*.

### Trial I: Global and specific evaluation of improvement

**Figure 1:**
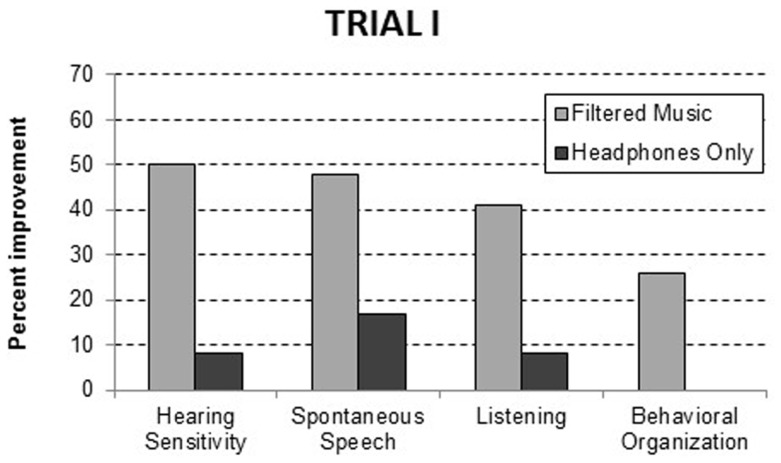
**Behavioral improvements at the 1-week post treatment assessment in Trial I**. The data are reported as the precent of participants with a specific behavioral problem who improved.

To evaluate the effectiveness of the filtered music treatment, group differences were evaluated with analyses of variance for each of the 10 behavioral dimensions included in the questionnaire. As illustrated in Figure 1, significant improvements, relative to the headphones only group, were noted in the filtered music group in hearing sensitivity, *F*(1, 29) = 6.46, *p* = 0.017; spontaneous speech, *F*(1, 49) = 5.61, *p* = 0.022; listening, *F*(1, 52) = 8.25, *p* = 0.006; and behavioral organization, *F*(1, 34) = 5.39, *p* = 0.027. The percent of the participants improving, who had a problem within each domain, is presented in Table 4. At 1-week post-intervention, analysis of variance confirmed that the filtered music group exhibited significantly more improvements summed across domains than the headphones only group (i.e., 2.36 versus 0.81), *F*(1, 62) = 7.76, *p* = 0.007.

**Table 4:** **Percent^a^ improving who had a problem within each behavioral domain at the 1-week follow-up**.

	Trial I		Trial II	
	Filtered music	Headphones only	Filtered music	Unfiltered music
Hearing sensitivity	**50^b^**, *n* = 18	8, *n* = 12	**43^c^**, *n* = 23	13, *n* = 16
Affect	19, *n* = 16	18, *n* = 17	25, *n* = 32	21, *n* = 19
Eye contact	41, *n* = 27	24, *n* = 17	33, *n* = 30	40, *n* = 20
Behavioral organization	**26^b^**, *n* = 19	0, *n* = 16	29, *n* = 28	18, *n* = 17
Emotional control	17, *n* = 18	0, *n* = 12	**24^c^**, *n* = 33	0, *n* = 19
Spontaneous speech	**48^b^**, *n* = 27	17, *n* = 23	51, *n* = 41	44, *n* = 25
Receptive speech	31, *n* = 26	9, *n* = 23	9, *n* = 45	15, *n* = 26
Listening	**41^b^**, *n* = 29	8, *n* = 24	30, *n* = 37	29, *n* = 21
Spontaneity	48, *n* = 25	20, *n* = 20	36, *n* = 22	36, *n* = 14
Relatedness	30, *n* = 30	13, *n* = 23	34, *n* = 32	29, *n* = 21

*^**a**^Defined by the number of individuals who improved divided by the number of individuals with problems (*n*) within the behavioral domain*.

*^**b**^Significant improvement relative to headphones only in Trial I*.

*^**c**^Significant improvement relative to unfiltered music in Trial II*.

### Trial II: Global and specific evaluation of improvement

**Figure 2:**
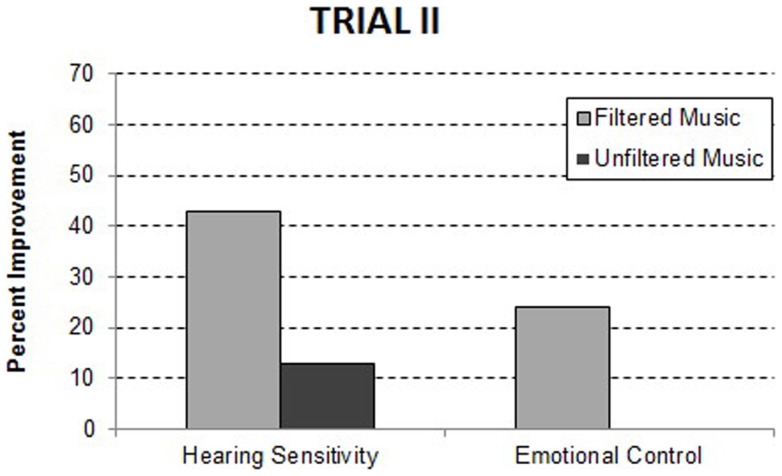
**Behavioral improvements at the 1-week post treatment assessment in Trial II**. The data are reported as the precent of participants with a specific behavioral problem who improved.

Since the relative benefits observed during Trial I could be attributed to listening to music, independent of the computer modulation of the acoustic features, Trial II was conducted contrasting the filtered music condition to the same music in an unfiltered form. The unfiltered music condition was similar to the “structured listening” condition described by Bettison (36). As illustrated in Figure 2, significant improvements in the filtered music condition relative to the unfiltered music condition were observed in both hearing sensitivity, *F*(1, 28) = 4.53, *p* = 0.040, and emotional control, *F*(1, 49) = 5.84, *p* = 0.019. The percent of the participants improving, who had a problem within each domain, is presented in Table 4. Note that when unfiltered music is used as the control, several of the benefits of filtered music condition observed in Trial I (i.e., spontaneous speech, listening, and behavioral organization) appear to be due to listening to music (i.e., unfiltered music) and not to the algorithm used to filter the music. Consistent with this interpretation, there was no significant difference in the sum of improvements for the filtered music group (1.98) when contrasted with the unfiltered music group (1.53). These data suggest that the unique benefit of the filtered music is a significant reduction in hearing sensitivity.

### Contrasts between Trial I and Trial II

Analyses of variance confirmed the similarity between the filtered music condition in Trial I and Trial II. The percent of participants improving on each domain was similar for the filtered music groups within Trial I and Trial II (see Table 4). Similarly, the number of problem domains was similar for all groups on entry into the protocol (see Table 3).

### Sharing behaviors

Video data from a random subsample of children in the filtered music condition (*n* = 61) were coded. The subsample was partitioned into three groups: children who had no hearing sensitivity at the start of the study (*n* = 34), children who showed improvements on hearing sensitivity following the intervention (*n* = 14), and children who had no improvements on hearing sensitivity following the intervention (*n* = 13). A repeated measures analysis of variance identified a significant group × condition interaction, *F* (2, 58) = 4.88, *p* < 0.011. Consistent with the parental reports, only the subgroup of children with improvement on hearing sensitivity increased the amount of sharing behavior during the 10-min semi-structured play-based protocol. Descriptive statistics are reported in Table 5. *Post hoc* Bonferroni adjustment confirmed that only children who were reported to improve on hearing sensitivity increased the amount of sharing behavior during the 10-min semi-structured play-based protocol following the 5-day program relative to the initial assessment (*p* < 0.005).

**Table 5:** **Hearing sensitivity (HS) and total number of shares (*N*, mean, and SD)**.

	*N*	Pre-intervention		Post-intervention	
		Mean	SD	Mean	SD
Children who improved on HS	14	5.71	7.31	9.86	10.53
Children who did not improve on HS	13	7.46	7.33	7.62	6.74
Children who had no HS	34	5.82	8.50	6.32	7.97

### Treatment effects on participants without hearing sensitivities

To investigate the effects of filtered music on the participants without hearing sensitivities, analyses of variance were calculated on each behavioral domain to identify possible behavioral domains that would improve in children without auditory hypersensitivities as a function of the filtered music. These analyses did not identify any specific behavioral domain that would reliably improve in the children without auditory hypersensitivities.

## Discussion

### Summary of findings and other research evaluating LPP

Two randomized controlled trials were conducted to evaluate the efficacy of LPP on auditory hypersensitivities and social behavior in children with ASD. Data from both trials confirmed that LPP (i.e., filtered music) selectively reduced auditory hypersensitivities. Trial I contrasted filtered music with a headphones only condition. The results of Trial I led to a more stringent Trial II in which filtered music was contrasted with an unfiltered music condition. In both trials, the LPP selectively reduced auditory hypersensitivities. In addition, within the filtered music groups the children with auditory hypersensitivities who improved following LPP significantly increased their spontaneous sharing behaviors. These findings, consistent with the polyvagal theory, support the hypothetical basis for designing the LPP as a neural exercise of pathways involved in regulating behavioral state, listening, looking, and other social engagement behaviors such as spontaneous sharing.

The current findings are consistent with a previous study (37), evaluating LPP with a more diverse sample of ASD children. In the previous study, the effectiveness of LPP was objectively assessed by evaluating auditory processing (assumed to be a function of the transfer function of the middle ear structures) and autonomic state (assumed to mediate behavioral state regulation). The study demonstrated that LPP significantly increased vagal regulation of the heart (i.e., increased amplitude of respiratory sinus arrhythmia) and normalized auditory processing on the filtered words and competing words subtests from the SCAN test for auditory processing disorder (38, 39). Collectively, the data from the current trials and Porges et al. (37) provide convergent preliminary support that LPP enhances function of the polyvagal “social engagement system” manifested in improved auditory processing, reduced auditory hypersensitivities, increased vagal regulation of the heart, and increased spontaneous social behaviors (e.g., sharing).

### Contrasts with traditional auditory intervention therapies

Since LPP delivers computer altered acoustic stimuli through headphones, it shares some of the features of auditory intervention therapies (i.e., AIT). However, although LPP is a “sound therapy,” it is not a traditional clinically available AIT (e.g., Ref. (40, 41)) and differs from these procedures in method and theory. First, LPP is based on the polyvagal theory and reflects a strategic attempt to engage neural regulation of specific structures involved in the social engagement system (28). Second, LPP focuses on auditory hypersensitivities that may be expressed by individuals with and without clinical diagnoses. Third, the effectiveness of LPP can be measured through well defined behavioral and physiological features of the social engagement system. Fourth, LPP was designed with several unique features to engage and to exercise the neural regulation of the middle ear muscles, including an understanding of the transfer function of the middle ear structures and the vulnerability of the fast twitch middle ear muscles to fatigue. Fifth, the duration of LPP is shorter (i.e., less than 5 h) than most forms of AIT. Therefore, the effects of LPP described in this study should not be generalized to any other form of auditory intervention.

There are several problems related to the evaluation of traditional auditory intervention therapies. First, since the interventions have evolved from clinical observations and insights, the neurophysiological theory underlying the interventions is often not well developed or tested. Second, research has been frequently structured to ask questions of efficacy instead of developing protocols to test theoretically relevant components of the treatment in order to understand the mechanisms and to refine the methodology. Third, since auditory interventions are applied within a clinical setting, several experimental design parameters are difficult to control including (1) a constant protocol, (2) limiting concurrent treatments including medication, (3) randomization of participants into conditions, and (4) the selection of outcome variables that are theoretically relevant to the intervention model. Perhaps the greatest limiting factor is the broad range of domains that auditory interventions are proposed to improve without a description of a causal link through which the intervention would result in functional changes in behavior. Due, in part, to the above limitations, the literature documenting an efficacy for the clinically available forms of AIT has been difficult to interpret.

Some studies evaluating the effectiveness of the AIT report improvements (42, 43) and others do not (36, 44–47). However, some of the above studies that do not support unique positive effects of AIT provide documentation of positive effects. For example, Bettison (36) reports positive effects in both the experimental group (received auditory training) and the control group (listened to the same unmodified music under the same conditions). Bettison suggests, consistent with our findings, that features in the AIT shared with listening to selected unmodified music may have beneficial effects on children with autism. Moreover, as our data suggest, if participants do not have auditory hypersensitivities, then the effects of LPP may be mediated through different biobehavioral pathways with unpredictable (i.e., non-specific) positive outcomes, which are not consistent with the middle ear transfer function model. Perhaps, similar to the outcomes with children without auditory hypersensitivities in the LPP trials, observed positive effects of AIT may be recruiting pathways outside of the middle ear model via the potential therapeutic calming effects of music and social support by clinicians.

Gilmor (48) conducted a meta-analysis based on several studies conducted in the 1980s with the Tomatis method involving 231 children. Gilmor clustered the outcome measures into five behavioral domains and identified small effects for linguistic, psychomotor, personal and social adjustment, and cognitive domains. Interestingly, he found no reliable effect in the auditory domain. These findings should be cautiously interpreted because the studies were limited by small sample sizes, issues related to defining control conditions, and limited use of random assignment. Regardless of these limitations, parents and clinicians of children with ASD have reported that forms of auditory intervention therapy have been helpful.

### Limitations of the current study

The data from the current study need to be cautiously interpreted for the reasons outlined below.

1. The major findings were dependent on the subjective reports of parents.
2. Some of the hypotheses tested were dependent on the small sizes of critical subgroups (e.g., individuals with or without auditory hypersensitivities who did or did not show improvements partitioned by the various treatment conditions).
3. The participants were receiving other treatments during the intervention and assessment period. Several participants were receiving daily interventions using behavioral approaches and other therapies, which may have enhanced or dampened the effects of the LPP.
4. Frequent contact of parents with therapists might bias parental reports and compromise the validity of the parents as objective informants. These factors could obfuscate the real effects of the intervention and inaccurately identify changes. Alternatively, features that might have improved could have been neglected. Possibly the hearing sensitivity domain on the parent questionnaire is less vulnerable to clinician–parent bias. Based on our experience, the therapists and parents appear to be less interested in this dimension, although it was the focal point of our study.
5. Improvements were observed in the groups not receiving the filtered music. Approximately 40% of the parents of children not receiving the filtered music reported improvements on at least one behavioral feature. These positive reactions might be due to non-specific features of the protocol, such as a relaxed intervention environment fostering social engagement and spontaneous play, as well as a positive “expectation” bias and the effects of familiarity with staff and context as the child progressed through the five laboratory sessions. However, the groups receiving filtered music diverged from the control groups when parents reported improvements in hearing sensitivity.
6. Standardized assessments of cognitive function and developmental landmarks were not evaluated. The lack of this information precluded confirmation of matching on these variables, although, based on the sample size, random assignment should have led to a reasonable expectation of matched samples. The randomization of participants, with regard to the evaluated parameters, was effective and there were no group differences in their representation. Standardized assessments of cognitive function and development would provide data to investigate two questions: (1) Are auditory hypersensitivities related to cognitive function and developmental landmarks? (2) Is the effectiveness of the LPP related to individual differences in cognitive function?
7. Our participants were young and on the severe end of the autism spectrum and the findings may not generalize to older or less severe ASD.
8. The studies precluded an opportunity to confirm the specific neural pathways responsible for the observed behavioral improvements. The methods employed could not confirm whether auditory hypersensitivity was due to a compromise in functional neural regulation of the middle ear muscles (as proposed by the polyvagal theory) and remediated through an exercise model.
9. The studies did not provide information necessary to distinguish among alternative pathways leading to or remediating auditory hypersensitivities, such as the potential influence of the intervention on damaged neural pathways (e.g., auditory or facial nerve), on damaged peripheral structures (e.g., middle ear and inner ear), or central structures involved in processing the acoustic signal or in cortical representation.
10. The hypothesized link between the middle ear transfer function and auditory hypersensitivities could be limited. Hypersensitivities, especially to high frequency sounds, might be due, not to the neural regulation of the middle ear muscles, but to the olivary cochlear reflexes. Tests of inner ear function and the degree of auditory hypersensitivity to high frequency sounds need to be evaluated to rule out this possibility.
11. The general improvements in behavior observed following a reduction in hearing sensitivity might not be related to the proposed integrative social engagement system. Rather, the enhanced behavior might be naturally occurring when the sounds are no longer painful and distracting.

### Future directions

A measure of the hypothesized intervening mechanism, the middle ear transfer function, has been missing from the formal experiments evaluating effectiveness of LPP. At the time the participants were tested, no commercial clinical or research device was available to monitor the middle ear transfer function. Without a sensitive measure of the middle ear transfer function, the only method to demonstrate efficacy was to quantify physiology, auditory processing, and measures of behavior and to infer that the LPP normalized an atypical middle ear transfer function. Recently we have developed a middle ear sound absorption system (MESAS) to measure the middle ear transfer function (49). MESAS provides an objective measure of the potential mediating role that middle ear muscles play in experiencing auditory hypersensitivities (see Ref. (50)).

By providing an objective measure of the middle ear transfer function, future research with MESAS will enable a selective test of the efficacy of LPP in normalizing the middle ear transfer function. If confirmed, LPP could be applied to individuals with atypical middle ear function including rehabilitation following otitis media. In addition, MESAS will enable future research to evaluate the behavioral and psychological consequences of an atypical middle ear transfer function, provide data to validate a quantitatively scaled measure of auditory hypersensitivities independent of subjective reports, and contribute to the improvement of interventions (e.g., LPP) that may function as efficient neural exercises to normalize the middle ear transfer function.

## Author Contributions

Stephen W. Porges was involved in all aspects of the research, including conception, design of the intervention stimuli, design of the protocol, analysis, interpretation, and writing the manuscript. Olga V. Bazhenova and Elgiz Bal were involved in the design, acquisition, and preliminary analyses and drafts. Nancy Carlson and Yevgeniya Sorokin were involved in acquisition of the data. Keri J. Heilman was involved in data acquisition, data analyses, and contributing to the final drafts. Edwin H. Cook was involved in developing the final draft and in interpreting the data. Gregory F. Lewis was involved in the development of the stimuli, developing the Middle Ear Sound Absorption System (MESAS), and in collecting and interpreting the preliminary data with MESAS.

## Conflict of Interest Statement

The authors declare that the research was conducted in the absence of any commercial or financial relationships that could be construed as a potential conflict of interest.
